# Annotations

**Published:** 1896-10-24

**Authors:** 


### Annotations.

### The Regimen of Early School Life.

Is it a usual thing in those little boys' schools which are conducted by ladies to begin school at nine in the morning, keep the children steadily at work until twelve, and then take them for a whole hour's walk, without giving them either luncheon or rest? The question is asked because at least one lady's school for little boys, which is by way of being a very superior school indeed, carries out this line of discipline with unflinching sternness. It should hardly be necessary to point out that such a method of training boys of the tender ages of six to nine is not consistent with normal growth, development, or health. To begin with, continuous lessons for three hours, at such an age, involve a very great mental and physical strain, and cannot but produce marked nervous fatigue. Then to add to this fatigue by insisting upon an hour's walk before any food is given is to further exhaust the nervous system, and to bring about a condition of impaired appetite and enfeebled digestion. All competent educationists have agreed with physiologists in this, that the first few years of school life should be made up largely of play. The three hours, from nine till twelve, for example, ought to be divided into at least three parts. At twelve o'clock, instead of a walk, luncheon should be given, or, at any rate, play for twenty minutes or half an hour, and then luncheon; but most certainly not a formal walk. Play, we repeat, should form a very great part of the education of little boys; and in no case should work be uninterrupted for long periods, or formal walks or other physical exercises be carried out when the children must, in the nature of the case, be beginning to be decidedly hungry.

### The Harveian Oration.

With all the usual ceremony, and before a large and distinguished audience, the Harveian Oration was delivered last Monday by Dr. J. Frank Payne at the Royal College of Physicians. It was very historical, very learned, and—if we may venture to whisper such a suggestion—a trifle dull. Nevertheless, in the midst of the scurry of modern life, it was well to be carried back to the past and to discuss the lives of the fathers of medicine as Dr. Payne discussed them, in such way as to assure ourselves again that they really lived, and although it was two thousand years ago, to see them again dissecting and investigating with the same energy and curiosity as we see around us in our own work to-day. Then, as to-day, there were coteries and parties which took opposite views; and then, as now, the theorist spoke at large with confidence unabashed, while the practical investigator buttonholed his friend and dragged him off to watch experiments, and for himself to see the truth—that *ignis fatuus* which, after two thousand years, we still run after, although, as our self-confidence assures us, with a trifle more success. What Dr. Payne did especially was to show that, with the spread of the renaissance of learning which had preceded the time of Harvey, not only had scholars gone back to the study of the Greek anatomists, whose works had been lost to Europe during the Dark Ages, and had in the end only reached Italy through a circuitous route by way of the Arabs, but that investigators had, for a hundred and fifty years before his time, been gradually adopting the teaching of Galen, and had gone "to Nature" to discover truth. To some it was no doubt disturbing to be told—and that in a Harveian Oration—that Harvey had not made his discoveries by dint of his own transcendent genius unassisted, but that, like other mortals, he had been swayed by the trend of the period in which he lived, and had built on the foundations laid by his predecessors. As Dr. Payne pointed out, however, something more than a mere raking among the fact heaps of nature is necessary for the making of a great discovery. Something allied to the poetic fancy is needed by the discoverer, a something by aid of which he sees in obvious facts relationships and interactions which, although plain enough when once pointed out, remain buried until they are deciphered by the imaginative faculty of genius. The facts as they existed in Harvey's time might be compared to the loose letters in a "letter puzzle." One solution of the problem was about as far as another from the right one, none was much better than the rest, until the master-mind of Harvey arranged the letters and spelled out the truth.

### Physically and Mentally Defective Children.

The Sub-Committee of the London School Board, which has recently dealt with the question of special schools for the education and training of physically and mentally defective children, has made certain recommendations that show how profoundly physiological considerations are modifying the ideas and influencing the practice of intelligent persons in this department of public activity. Among other facts the committee, through the Board, suggest to their lordships "that experience has shown that in several cases the dormant faculties of children excluded from public elementary schools on the ground of imbecility have been aroused by the teaching given in the special centre, and would urge that some period of probation should be permitted before any child is condemned to the confinement of an asylum." If physiology teaches one thing more than another it is this: that sanity and insanity, mental capacity and imbecility, morality and criminality, are terms of extraordinary elasticity; and that one and the same person is often capable in early life of being so influenced by the forces of environment as to become either a fairly capable citizen or an imbecile, either a responsible member of the community or a degraded criminal. What is needed to give the teachings of physiological science fair play is undoubtedly a special institution, or institutions, officered by specially intelligent and specially kind men and women. To make responsible citizens out of children who but for special education and training would become hopeless imbeciles and criminals, is surely an object worthy of the highest science and the worthiest religion of the times. By all means let us have an adequate number of "special institutions," and let the recommendation of the London School Board, that "some period of probation shall be permitted before any child is condemned to the confinement of an asylum," be forthwith adopted as a working rule by every school board in the kingdom.

